# Integrating Abnormal Gait Detection with Activities of Daily Living Monitoring in Ambient Assisted Living: A 3D Vision Approach

**DOI:** 10.3390/s24010082

**Published:** 2023-12-23

**Authors:** Giovanni Diraco, Andrea Manni, Alessandro Leone

**Affiliations:** National Research Council of Italy, Institute for Microelectronics and Microsystems, SP Lecce-Monteroni km 1.200, 73100 Lecce, Italy; andrea.manni@imm.cnr.it

**Keywords:** Abnormal Gait Detection, Ambient Assisted Living, RGB-D Camera, Temporal Convolutional Network, Gated Recurrent Unit, Long Short-Term Memory Autoencoder

## Abstract

Gait analysis plays a crucial role in detecting and monitoring various neurological and musculoskeletal disorders early. This paper presents a comprehensive study of the automatic detection of abnormal gait using 3D vision, with a focus on non-invasive and practical data acquisition methods suitable for everyday environments. We explore various configurations, including multi-camera setups placed at different distances and angles, as well as performing daily activities in different directions. An integral component of our study involves combining gait analysis with the monitoring of activities of daily living (ADLs), given the paramount relevance of this integration in the context of Ambient Assisted Living. To achieve this, we investigate cutting-edge Deep Neural Network approaches, such as the Temporal Convolutional Network, Gated Recurrent Unit, and Long Short-Term Memory Autoencoder. Additionally, we scrutinize different data representation formats, including Euclidean-based representations, angular adjacency matrices, and rotation matrices. Our system’s performance evaluation leverages both publicly available datasets and data we collected ourselves while accounting for individual variations and environmental factors. The results underscore the effectiveness of our proposed configurations in accurately classifying abnormal gait, thus shedding light on the optimal setup for non-invasive and efficient data collection.

## 1. Introduction

Gait disorders, which are common among the elderly population, pose a significant threat to their overall well-being. They often lead to balance problems, injuries, disabilities, and loss of independence, ultimately diminishing quality of life [1].

These disorders are closely associated with musculoskeletal and neurological impairments, making them some of the primary underlying causes of an abnormal gait. Remarkably, studies indicate that around 25% of individuals aged 70 to 74 exhibit gait irregularities, a figure that nearly doubles to almost 60% for those in the 80 to 84 age bracket [2]. The identification and characterization of gait anomalies play a pivotal role in diagnosing these musculoskeletal and neurological disorders. Gait analysis has emerged as a valuable method for assessing these conditions; however, it is fraught with challenges. One of the primary challenges lies in the absence of universally accepted standards for evaluating the gait of older individuals [3]. 

Some researchers focus on fundamental time–distance factors, like walking speed and step length. Others delve into intricate biomechanical analyses by examining various parameters, such as joint rotation and position, to uncover musculoskeletal and neurological irregularities [4]. Regardless of the chosen methodology, the process of manually analyzing gait data involves a labor-intensive phase, wherein relevant gait parameters are derived through visual inspection and annotation. The accuracy of this manual analysis is significantly dependent on the expertise of healthcare professionals who perform the assessment. Specifically, this refers to the meticulous extraction of gait features from raw data, a task that is time-consuming and subject to potentially subjective interpretations [5]. Recognizing these challenges, recent advancements in machine learning (ML) and deep learning (DL) have demonstrated their effectiveness in automating the diagnosis of musculoskeletal and neurological disorders, thereby reducing the burden on healthcare personnel and ensuring the reliability of diagnostic outcomes [6].

Cimolin et al. [7] and Ferraris et al. [8] tested an RGB-D sensor’s ability to assess gait in neurological diseases. They compared it to a 3D instrumented gait analysis system while focusing on spatio-temporal parameters and center of mass excursion during gait. The RGB-D system, using a custom step segmentation algorithm, yielded similar results to the 3D gait analysis system. However, differences in reference points and algorithms may impact direct result comparisons. Kaur et al. [9] introduced an innovative DL framework for predicting gait dysfunction in multiple sclerosis and Parkinson’s disease using multi-view visual data. Their system offers a cost-effective and convenient remote monitoring tool for classifying neurological gait disorders. It is contactless, thus eliminating the need for certified professionals, and it can assess the gait of older adults in real-world settings. Their open-source workflow is accessible on GitHub. Unlike other studies, they compared their approach with 16 diverse models, examined various designs, and automated feature engineering. They also focused on the interpretability of optimal models, a facet missing in previous research. Furthermore, their ternary classification approach shows promise for identifying different origins of neurological gait disorders with similar performance to binary classification approaches.

Tang et al. [10] developed an affordable, portable, vision-based gait analyzer that uses pose estimation to extract various spatiotemporal gait parameters from Timed-Up-and-Go (TUG) videos. These parameters include step length, gait speed, step frequency, step width, standing up time, and turning back time. They tested the accuracy of this analyzer against experienced neurologists and found it to be comparable. They utilized the AlphaPose algorithm for precise pose recognition and a double-threshold signal detection method to minimize errors from body movement during walking. The study also demonstrated that their supervised ML classifier effectively detected abnormal gait patterns when compared to human assessments. To train binary classifiers for detecting abnormal walking patterns, they employed three classical ML models: Naive Bayes (NB), Logistic Regression (LR), and Support Vector Machine (SVM) algorithms. The measurement data were rigorously tested for normality using the Shapiro–Wilk normality test. The study involved a total of 404 participants with TUG videos, with 120 participants excluded based on predefined exclusion criteria, resulting in 284 participants finally included in the analysis.

In the study by Jun et al. [11], the authors used Kinect v2 skeleton data and a multilayered Gated Recurrent Unit (GRU) classifier to classify various pathological gaits. They also determined the relevance of different joints for this classification. The GRU is an advanced Recurrent Neural Network (RNN) architecture comparable to Long Short-Term Memory (LSTM), and, in their research, the GRU classifier outperformed the LSTM classifier by 2.88% in terms of classification accuracy, indicating that the proposed GRU structure is more suitable and robust for classifying pathological gaits using skeleton-based gait data.

Guo et al. [12] developed a mobile 3D gait analysis system that uses an RGB-D camera to track and analyze human gait in real time. This system is designed for gait monitoring at home in normal living conditions, particularly for the elderly and individuals with neurological conditions, who are at a higher risk of falls due to gait disorders. The main aspects of the system include view-invariant 3D lower limb pose estimation, lower limb skeleton tracking using Simultaneous Localization and Mapping (SLAM), extraction of gait parameters (i.e., joint velocities, gait speed, step/stride length, 3D joint trajectory), and abnormal gait detection using SVMs and bidirectional LSTM networks. The system’s robustness was validated with data from healthy volunteers, and it outperformed previous methods in recognizing different gait patterns. The integration of SLAM and pose estimation improves the accuracy of each subsystem.

One notable common aspect across the reported papers is the use of supervised machine learning (ML) and deep learning (DL) approaches for extracting gait features in the context of anomaly detection and classification. Many of these studies involve laboratory settings with healthy subjects simulating pathological gaits in order to train supervised models. However, gait abnormalities are subject-specific; thus, models trained only on simulated abnormalities might not guarantee the same performance achieved in a laboratory setting when tested in the field with unhealthy subjects [13].

The challenges posed by the significance of subject-specific differences were underscored by Guo et al. [13]. They highlighted the necessity for unsupervised learning to rectify the issue of poor generalization when applied to novel subjects. To tackle these challenges, the authors introduced an approach called Maximum Cross-Domain Classifier Discrepancy (MCDCD). This method was designed to alleviate domain discrepancies, thereby enhancing the detection of gait abnormalities through Unsupervised Domain Adaptation (UDA). The UDA method leveraged information gleaned from labeled training subjects to optimize classification performance when applied to a previously unseen test subject.

Chen et al. [14] highlighted the multifaceted advantages associated with the analysis of gait within the context of activities of daily living (ADLs). This analytical approach proves instrumental in various aspects, including early disease diagnosis, the identification of potential health concerns, and the ongoing monitoring of treatment efficacy. Utilizing Parkinson’s Disease (PD) as an illustrative example, it is noteworthy that the early stages of PD may manifest with subtle symptoms and inconspicuous locomotor or balance challenges. As emphasized by the authors, scrutinizing gait performance during diverse ADLs allows for the efficient extraction and analysis of specific gait parameters, such as turning steps, turning time, and turning angle, thereby enhancing the diagnostic capabilities for PD. Beyond the realm of gait parameters, this approach yields invaluable insights into mobility—an essential consideration for clinical applications. Continuous monitoring of ADLs provides a dynamic assessment of mobility changes throughout the day and week, thus offering a nuanced understanding of responses to interventions and the influence of environmental factors on mobility. Accumulated research underscores that routine assessment of everyday mobility serves as a pertinent indicator of disease progression and rehabilitation effectiveness. Furthermore, the sequential analysis of various ADLs over time emerges as a powerful tool for unearthing potential health issues.

Climent-Perez et al. [15] pointed out the potential benefits of recognizing ADL from video for active and assisted living. They emphasized the need to tap into the full potential of this technology, which can aid behavior understanding and lifelogging for caregivers and end users. Their paper introduced alternative pre-processing techniques, specifically for the Toyota Smarthomes dataset, to enhance action recognition. These techniques involved normalizing skeletal pose data and expanding activity crops for RGB data. Their results showed that the proposed techniques improved recognition accuracy, especially when combining pre-trained branches and feeding outputs into the separable spatio-temporal attention network. However, the attention network’s contribution to overall improvement was marginal.

Beyond the previously mentioned advantages, the integration of gait analysis into the realm of ADL monitoring unveils novel potential. This integration facilitates the smooth blending of supervised postural descriptors commonly used for ADL classification with unsupervised approaches for anomaly detection. Such a cohesive synthesis holds the promise of surpassing the limitations inherent in fully supervised methods, especially when dealing with subject-specific patterns that may prove challenging for conventional approaches.

To the best of the authors’ knowledge, there are no contributions in the literature where the issue of abnormal gait detection has been explored in conjunction with ADL monitoring within Ambient Assisted Living (AAL) applications.

## 2. Materials and Methods

**Figure 1:**
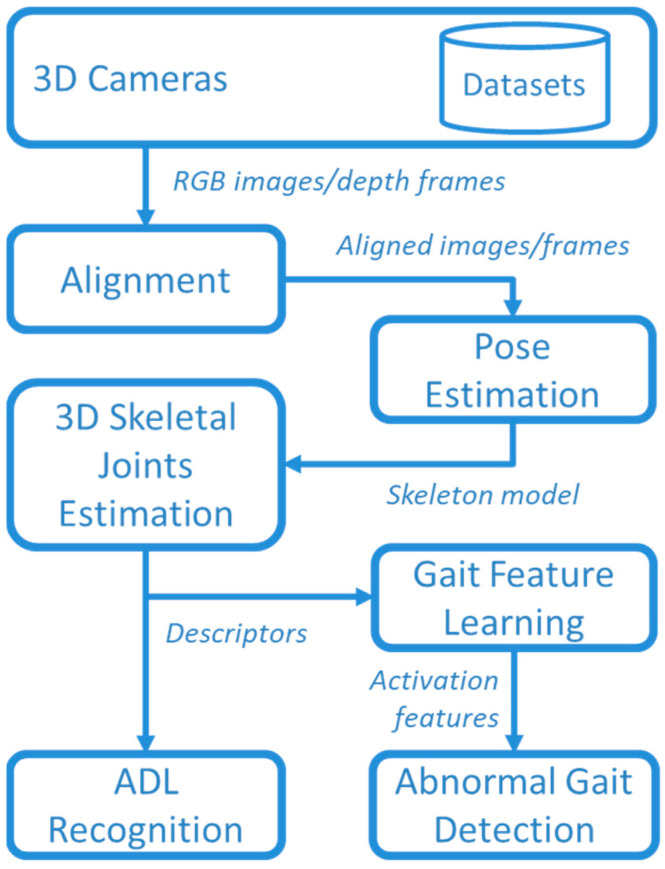
Overview of the whole framework.

The aim of this study was monitoring the human gait during the execution of common ADLs. The suggested framework for ADL recognition and gait monitoring is presented in Figure 1. Data were collected using two 3D cameras in a laboratory setting, where several volunteers performed various ADLs together with “normal” and “abnormal” gaits, as detailed in Section 2.1. Data captured by 3D cameras were stored in datasets for offline analysis, and, in addition, publicly available datasets were used for comparison purposes (as better specified in Section 2.1). 

RGB images and depth frames provided by the 3D cameras were aligned to create a comprehensive understanding of the scene. This alignment process allowed for the integration of color information with spatial data obtained from the depth frames. By accurately aligning RGB images and depth frames, it became possible to create a synchronized representation where each pixel in the RGB image corresponded to a specific point in the 3D space with a known depth value. In this way, a 3D representation of joints was obtained by using the MediaPipe Pose library, as detailed in Section 2.2. 

The 3D skeletal descriptor was used for both ADL recognition and abnormal gait detection. The recognition of ADLs was based on supervised DL by comparing Temporal Convolutional Network (TCN) and GRU approaches, described in Section 2.3 and Section 2.4. Furthermore, the unsupervised approaches were used for DL gait features that, in turns, were used for detecting abnormal gaits with an autoencoder (AE) based on bidirectional LSTM, as described in Section 2.5.

### 2.1. Data Collection and Experimental Setup

**Figure 2:**
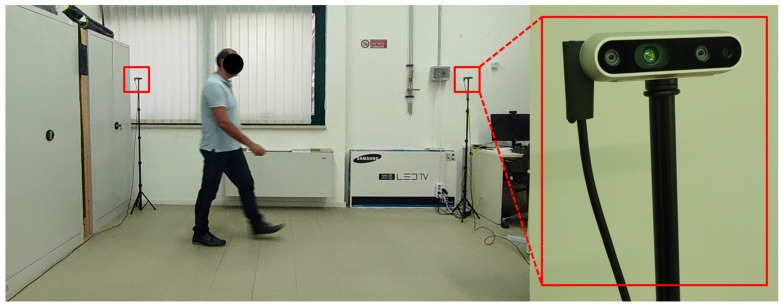
Laboratory setup for data collection.

**Figure 3:**
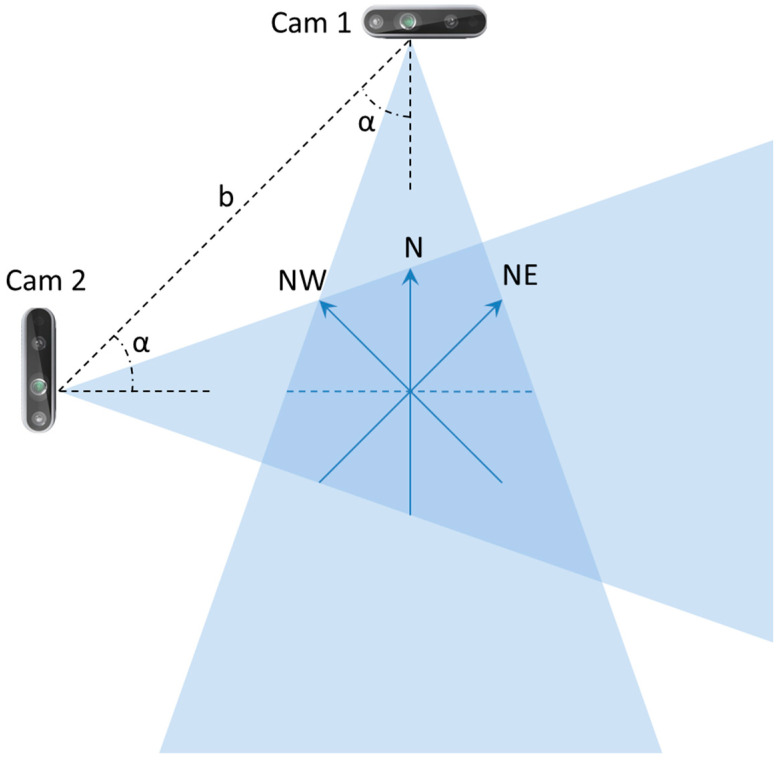
Schematic representation of the camera setup used in the laboratory setting.

Because the aim of this study was to explore the detection of abnormal gaits during monitoring of ADLs, specific data collection was conducted in the laboratory setting shown in Figure 2. Three male volunteers were involved (age 45 ± 5 years, height 180 ± 10 cm), and they performed the five basic ADLs of Eating (EAT), Dressing (DRS), Drinking (DRN), Sleeping (SLP), Walking (WLK), and one instrumental ADL (IADL) of Housekeeping (HSK), i.e., sweeping the floor. All activities were performed in three different directions (NE, N, NW) inside the intersection of the two FOVs, as shown in Figure 3. In addition, all activities were repeated at different heights h of the two cameras, and the activities WLK and HSK were performed in “normal” and “abnormal” ways. 

More specifically, the abnormal execution was simulated when moving from one position to another or getting out of bed or chairs by adopting the following expedients: (1) unnaturally slowed movements, (2) limped walking, and (3) stopping suddenly and starting again several times. Abnormal gaits were primarily involved in walking and housekeeping activities. In addition, the simulation of abnormal execution also occurred during transitions between positions and activities, such as getting out of bed or chairs.

In order to achieve a realistic simulation of abnormal gait, participants underwent a guided process based on specific guidelines provided by neurologists with expertise in neuromotor disorders, specifically targeting Parkinson’s and Huntington’s diseases. These guidelines were meticulously crafted to replicate the distinctive gait patterns associated with these neurodegenerative disorders. The guidelines centered on several critical aspects integral to the manifestation of abnormal gait. Specifically, participants were instructed to pay attention to and simulate abnormalities related to left–right asymmetry, tremor, rigidity, and postural instability. Each of these components represents key characteristics observed in individuals affected by neuromotor disorders. 

Left–Right Asymmetry: Participants were directed to simulate variations in stride length, step time, and overall movement coordination between the left and right sides. This aimed to capture the asymmetrical gait commonly observed in individuals with neuromotor disorders.

Tremor: Emphasis was placed on reproducing the rhythmic and involuntary shaking movements associated with tremors. Participants were guided to incorporate tremor-like features into their gait patterns to authentically represent this characteristic aspect of abnormal motor function.

Rigidity: The guidelines addressed the simulation of increased muscle tone and stiffness characteristic of rigidity in neuromotor disorders. Participants were instructed to convey a sense of resistance and inflexibility in their movements, thus mirroring the restricted mobility often seen in affected individuals.

Postural Instability: Participants were guided to mimic challenges in maintaining balance and stability during walking. This involved incorporating swaying or unsteadiness into their gait, thus replicating the postural instability commonly observed in individuals with neuromotor disorders.

By providing detailed guidelines that specifically targeted these nuanced aspects of abnormal gait, we aimed to ensure a comprehensive and faithful representation of the distinctive motor characteristics associated with neuromotor disorders. This approach facilitated the creation of a simulated abnormal gait dataset for effective abnormality detection analysis.

The previously mentioned parameters (cameras’ heights h, baseline b, orientation angles α, and subjects’ orientations during ADL execution) are summarized in Table 1.

The process of selecting the six activities was driven by a deliberate consideration of privacy concerns and ethical implications associated with the monitoring of specific ADLs. Notably, certain activities, such as toileting and bathing, if monitored using a camera, could potentially infringe on privacy. To address this concern, our objective was to strike a balance between achieving a comprehensive representation of daily activities and upholding ethical considerations. In the context of our study objectives, the inclusion of housekeeping activities (in particular, the task of sweeping the floor) was purposeful. This choice was motivated by the inherent physical movement involved in the activity (namely, walking), which presents a conducive scenario for effective abnormality detection through continuous monitoring. The incorporation of this specific housekeeping activity contributes to the diversity and relevance of the selected activities for our research objectives.

For the laboratory data collection, two depth cameras, Intel^®^ RealSense™ depth camera D435i (© Intel Corporation, Santa Clara, CA, USA), were used, as depicted in Figure 2, and for which the technical specifications are summarized in Table 2. For each volunteer, 72 RGB-D sequences lasting 1 min were collected. Each sequence included 1440 frames, providing a total of 103,680 frames for each volunteer. Each ADL class consisted of 17,280 RGB-D frames, resulting in 576 data samples per ADL, with a frame rate of 24 fps and a window size of 30 frames. Furthermore, to train models described below, the dataset was partitioned into training, validation, and testing sets with proportions of 70%, 15%, and 15%, respectively.

The furniture included in the simulation comprised a bed, a chair, and a table. For the specific activities of EAT and DRN, participants were seated on a chair in front of a table. The DRS activity involved sitting on the bed, while the SLP activity was performed lying on the bed. The housekeeping activity entailed sweeping the floor with a broom. Regarding the consistency of activities, it is important to note that each activity involved the continuous repetition of simple movements throughout the 12 min duration (1 min sessions with 12 repetitions). For example, in the case of housekeeping, the action of moving around while swinging the arms holding a broom was consistently repeated. This repetitive nature facilitates activity recognition, so capturing a few frames is sufficient to identify the type of activity.

To compare the suggested framework with the state of the art, publicly available data were also considered, i.e., the Pathological Gait Datasets provided by Jun et al. [11]. The goal of the authors was to classify complicated pathological gait patterns, including both normal gaits and five pathological gaits: antalgic, stiff-legged, lurching, steppage, and Trendelenburg gaits. The datasets used in the study were collected using a multiperspective Kinect system composed of six cameras arranged in two rows along the subject’s direction of walking. Ten healthy individuals participated in data collection by simulating the five pathological gaits based on provided guidelines. The Kinect sensors (© Microsoft, Redmond, WA, USA) captured 3D coordinates of 25 joints, resulting in a total of 10 participants, 6 gait types per participant, and 20 instances per type, which is equal to 120 instances for each gait.

### 2.2. Estimation of 3D Skeletal Joints

**Figure 4:**
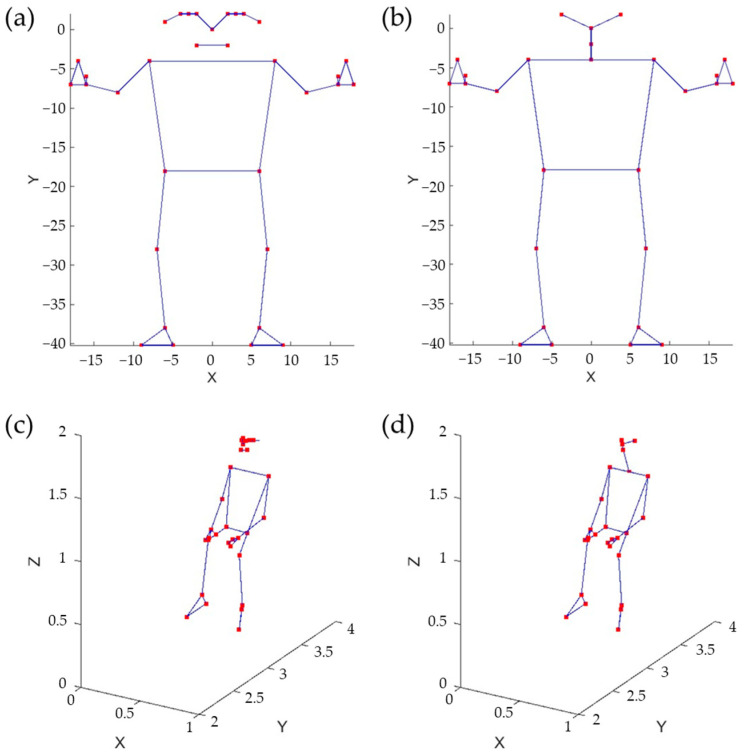
Skeleton models, original (**a**) and reduced (**b**). Sitting pose represented using the original model (**c**) and the reduced one (**d**).

The body pose was captured using the MediaPipe (© Google, Mountain View, CA, USA) Pose Landmarker [16], which tracks 33 body landmark locations representing the approximate location of the head, torso, and limbs. The skeleton model of the human body provided by MediaPipe is shown in Figure 4a. To obtain effective features, it is crucial to utilize a fully connected skeleton graph. Therefore, the original MediaPipe skeleton model was reduced, as illustrated in Figure 4b. As an example, Figure 4c,d depict body skeletons captured using both models.

Various skeletal data representation approaches were considered. A skeleton, in this context, is a graph characterized by N vertices and N−1 edges. The skeletal graph can be depicted using three different methods. The first method involves listing the 3D Euclidean coordinates of its vertices, necessitating the use of 3 × 27 parameters ($x_{n},\;y_{n},\;z_{n}$). The second method entails creating an adjacency list of Spherical coordinates for the vertices. Each vertex’s Spherical coordinates are determined concerning a Spherical reference system, with its origin located at the previous vertex in the list. This method also requires 3 × 27 parameters ($\rho_{n},\;\phi_{n},\;\psi_{n}$). The third method uses an adjacency list of 3D rotations to define each vertex in relation to the preceding adjacent vertex in the list. Note that in the second and third cases, it is possible to assume a fixed distance between adjacent vertices, which results in a reduction in the total number of parameters.

### 2.3. The Temporal Convolutional Neural Network

TCN networks, as elucidated by Bai et al. [17] in their empirical study, represent convolutional neural networks engineered for the purpose of effectively analyzing time series data. In comparison to LSTM networks, TCN networks exhibit superior performance attributes. A salient characteristic of TCN networks resides in their incorporation of dilated causal convolutions (DCCs), wherein only temporal values preceding the current time step are considered. This strategic temporal convolution enables TCN networks to capture extensive long-term temporal patterns, thereby augmenting the receptive field without necessitating the utilization of pooling layers. Consequently, this design choice mitigates any adverse effects on spatial resolution, as emphasized by Lara et al. [18] in their work on temporal modeling.

Given the input sequence $x\in R^{N,M}$, the dilation factor d, and the convolutional kernel g of size S∈N (with N>S>d), the DCC with dilation factor d at the time instant i is defined as follows:

(1) $${DCC}_{d}\left(x,g\right)(i)=\sum_{j=0}^{S-1}g\left(j\right)x_{i-dj}$$

that, for d=1, corresponds to the classical convolution. By exponentially increasing the dilation factor at each layer, it is possible to obtain a wider receptive field. In this way, considering a total amount of K layers, the size D of the receptive field of the network is given by

(2) $$D=\left(S-1\right)\left(2^{K}-1\right)+1.$$

**Figure 5:**
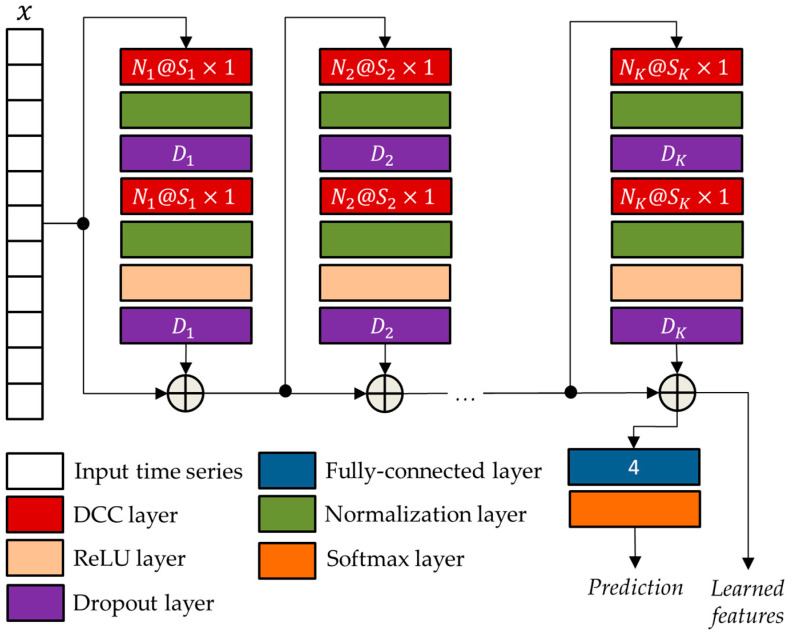
The general TCN architecture with K residual blocks.

The general TCN architecture, provided in Figure 5, has a modular structure based on K residual blocks, each including two DCCs with equal dilation factor, depth, and size. Such blocks are characterized by residual connections that, as suggested by He et at. [19], improve the performance of deep architectures by adding the block input to its output.

The network parameters of the TCN architecture were optimized using the genetic approach presented by Diraco et al. in [20]. For this purpose, a variable number of residual blocks is considered ranging from 3 to 5, with each block consisting of two normalization layers, two dropout layers, a DCC layer, and a rectified linear unit (ReLU) [21]. The optimized parameters obtained at the end of the optimization process, i.e., the numbers of convolutional filters $N_{k}$, the filter sizes $S_{k}$, and the drop out percentages $D_{k}$, are reported in Table 3.

### 2.4. The Gated Recurrent Unit Neural Network 

The GRU, a pivotal innovation in the realm of RNNs, was originally introduced by Cho et al. [22] in the context of statistical machine translation. Unlike its predecessor, the LSTM, the GRU offers similar capabilities for data control without the need for additional memory units. This strategic design choice allows the GRU to effectively address the issue of gradient vanishing that often plagues traditional RNNs, thereby facilitating the learning of intricate mappings from one sequential piece of data to another.

The GRU stands out from conventional RNNs by incorporating a reset gate and an update gate within a unified update mechanism. These gates enable the network to selectively determine what information to discard and what to retain from prior hidden states, thereby mitigating issues related to the loss of relevant information over long sequences. Consequently, the GRU is well-equipped to tackle long-term dependencies inherent in various time series applications, thus extending its influence beyond machine translation to other domains, such as speech recognition and healthcare.

**Figure 6:**
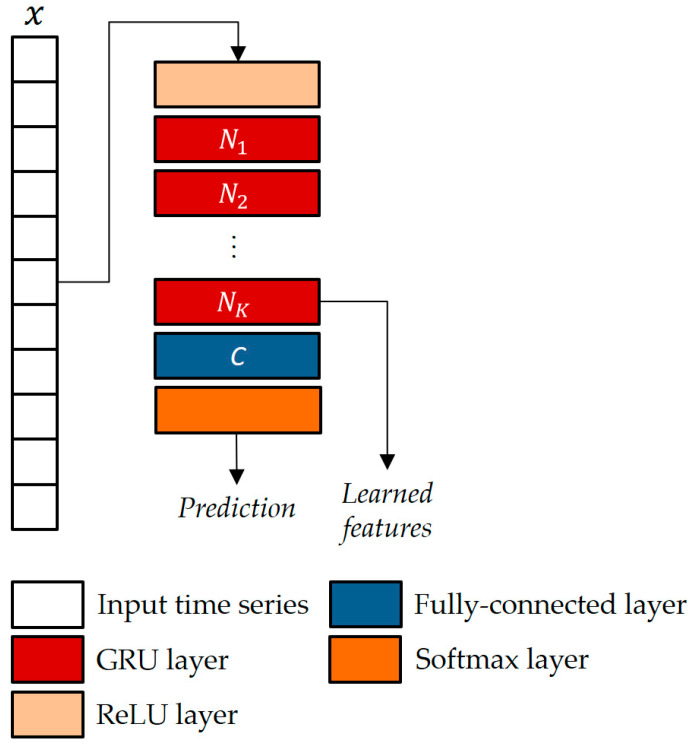
The GRU-based classifier architecture with K units and C classes.

In this study, the GRU architecture suggested by Jun et al. [11] was used, as depicted in a generalized format in Figure 6. In a GRU, the way the current hidden state is influenced differs from a standard RNN. Instead of a direct influence, the GRU employs two gates: an update gate to control the amount of past information to consider and a reset gate to regulate the amount of information to forget. More formally, given $x_{k}$ at the time step k, the candidate state $h_{k}$ is as follows:

(3) $$h_{k}=\left(1-z_{k}\right)*h_{k-1}+z_{k}*tanh(W*\left[r_{k}*h_{k-1}\;\;\;\;x_{k}\right]+b)\;,$$

where $z_{k}$ is the gate, activated through a sigmoid function σ(·):

(4) $$z_{k}=\sigma (W^{\prime }*\left[h_{k-1}\;\;\;x_{k}\right]+b\prime )\;,$$

and $r_{k}$ is the reset gate, also activated via sigmoid function:

(5) $$r_{k}=\sigma \left(W^{\prime \prime }*\left[h_{k-1}\;\;\;x_{k}\right]+b\prime \prime \right)\;.$$

When an entry in $z_{k}$ is approaching 1, it means that the current state heavily relies on the candidate state. Conversely, if an entry is approaching 0, then the current state leans more on the previous state. In simple terms, $z_{k}$ essentially decides the proportion of the candidate state that should be incorporated into the current state. In Equations (3)–(5), $W,\;W^{\prime },W\prime \prime$ and $b,b^{\prime },b\prime \prime$ represent the weights and biases vectors, respectively.

The parameters of the GRU architecture were chosen as in [11], i.e., k=4 blocks and $N_{k}=125$ hidden neurons.

### 2.5. The Autoencoder Hybrid Model Based on Long Short-Term Memory

To increase the representation power of learned features, the two supervised models, i.e., TCN and GRU, were combined with a (bidirectional) LSTM autoencoder (LSTMAE).

**Figure 7:**
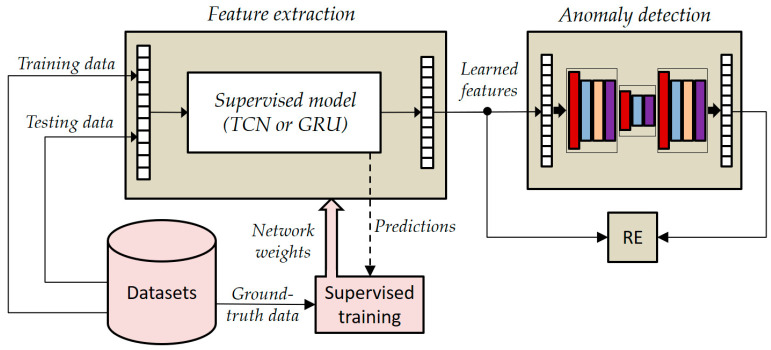
General overview of the joined architecture based on LSTMAE.

In the joint architecture, the supervised networks played the role of feature extraction, while the LSTMAE network played the role of anomaly detector. As shown in Figure 7, the supervised models were pre-trained by using collected time series datasets, whereas ground-truth data were used for performance evaluation. The ground-truth data were gathered through a manual approach. This involved annotating various elements, such as the performed activities and associated geometric parameters, including person orientation and camera heights, during the data collection process. 

It is crucial to emphasize that the volunteer who participated in the training phase of the LSTMAE network was intentionally not engaged in the pre-training of the supervised models. This deliberate separation of roles aims to closely mirror real-world scenarios, where the individuals being monitored typically do not partake in the training of supervised models. This approach ensures that our experimentation aligns more faithfully with practical conditions and enhances the applicability of our findings to real-life situations.

In the testing phase, instead, the joined networks operate in an unsupervised mode because activations (i.e., learned features) extracted from the pre-trained TCN/GRU are supplied as input to the LSTMAE, which operates naturally in an unsupervised manner, and then the reconstruction error (RE) is estimated by comparing learned features and reconstructed ones using the following equation:

(6) $$RE\left(z,\hat{z}\right)=\frac{1}{2}\sum_{i=1}^{M}{\left\|z_{i}-{\hat{z}}_{i}\right\|}^{2}\;,$$

where $z_{i}$ is the time series provided as input to the AE network, $E\left(z_{i}\right)$ is the encoded representation provided by the encoder network, and ${\hat{z}}_{i}=D\left(E\left(z_{i}\right)\right)$ is the reconstructed input provided by the decoder. In such a way, the LSTMAE can be trained on the “normal” activity (i.e., walking, because we are interested in abnormal gait detection) as performed by the monitored subject, not by other healthy subjects as happens in a supervised scenario.

**Figure 8:**
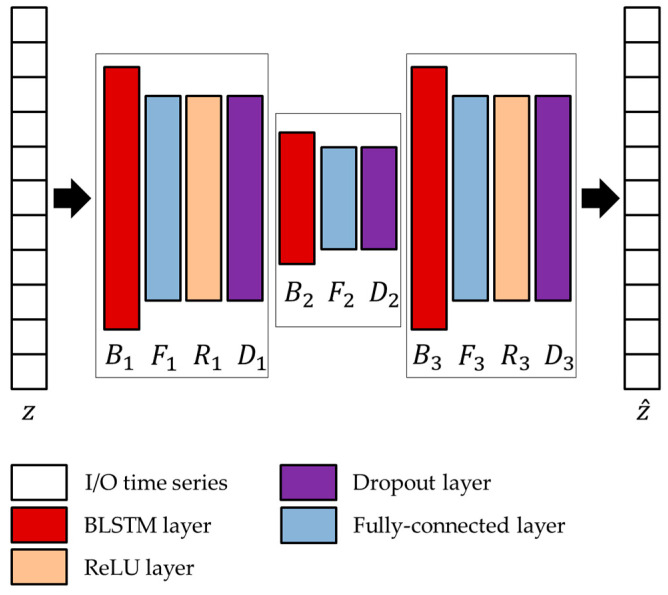
Architecture of the LSTMAE network adopted in conjunction with the two unsupervised models.

In the joint architecture, the parameters of the LSTMAE network are optimized based on the activations extracted from the TCN/GRU networks and by following the approach presented in Diraco et al. [20]. The optimized architecture is provided in Figure 8, and optimized network parameters, i.e., number of hidden units $B_{k}$, output size $F_{k}$, and dropping out probability $D_{k}$, are reported in Table 4 and Table 5.

As already mentioned, the LSTMAE was trained using a semi-supervised approach. This was accomplished by observing normal walking patterns and subsequently identifying abnormal walking patterns characterized by significant deviations leading to high reconstruction errors. It is important to note that normal walking patterns could be exhibited by the subjects under observation or replicated by other individuals in a controlled setting, such as a laboratory. In this study, both scenarios were thoroughly explored by training the model LSTMAE with data from various sources. 

The data from subjects different from the monitored individual were designated as “inter-class samples”, thus representing simulated normal walking patterns. Conversely, data obtained from the same monitored subject were categorized as “intra-class samples”, signifying instances where their normal walking patterns were directly observed.

## 3. Experimental Results

In this section, the results of the laboratory experiments are presented. First of all, the three skeleton data representations discussed in the previous section were compared in terms of processing speed.

An evaluation of these three representation approaches was conducted in terms of processing time. It was found that the second approach was the best performing, as it took only 17% of processing resources for feature extraction. In contrast, the first approach took 43%, and the third one even took 63%. Thus, the second approach was employed in the experiments, assuming a constant distance between consecutive joints (i.e., a feature space of 54 dimensions).

The data collection software was entirely written in Python 2.7 using the wrapper for Intel RealSense SDK 2.0, pyrealsense2 [23], which provides the C++ (Visual Studio 2017) to Python binding required to access the SDK. Meanwhile, as already mentioned, the MediaPipe Pose library (version 0.10.10) was used for the body skeleton capture. The data collection software was executed on an MS-Windows-OS (© Microsoft, Redmond, WA, USA)-based mini-PC with a Core i5-9600T 2.3 GHz Intel (© Intel Corporation, Santa Clara, CA, USA) processor and 8 GB of RAM. In contrast, the performances of the TCN, GRU, and LSTMAE models were evaluated in Matlab R2023b (Natick, MA, USA) using the Deep Learning Toolbox (version 23.2) executed on an MS-Windows-OS-based workstation equipped with two NVIDIA GPUs (Santa Clara, CA, USA), RTX 3060 (Ampere), and Titan X (Pascal), both with 12 GB GRAM. 

The data supplied to the Deep Neural Network (DNN) models consisted of time series comprising skeleton pose features, as per the previously established discussions. Concerning the temporal extent of the time series furnished at each computational iteration, it was fixed at N=30 frames, wherein each frame was characterized by a dimensionality of M=54. These specifications were chosen to ensure a consistent frame rate of 20 fps during the data acquisition phase, a minimum requisite to facilitate the near-real-time operation of ADL monitoring applications [24]. It is important to note that this frame rate value does not encompass the computational load incurred by DNN processing, but only camera RGB-D frames grabbing, 3D point cloud computing, skeleton pose capture, and feature extraction.

**Figure 9:**
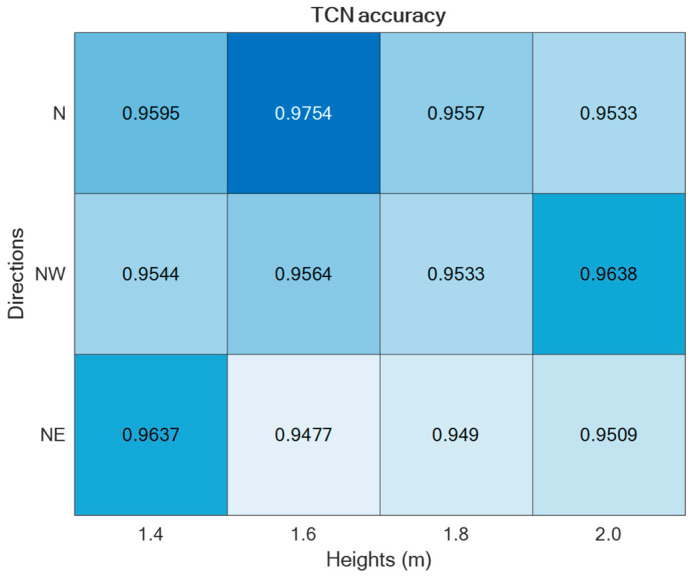
ADL recognition accuracies upon varying the parameters h and d by using the TCN-based supervised approach.

**Figure 10:**
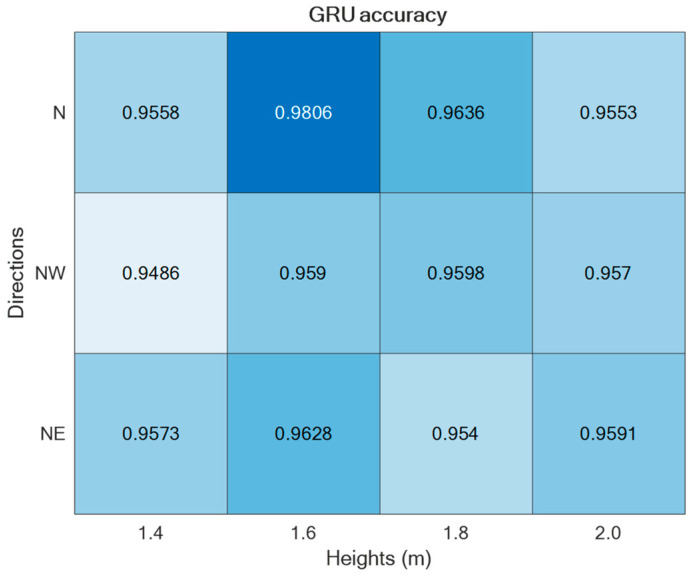
ADL recognition accuracies upon varying the parameters h and d by using the GRU-based supervised approach.

The two supervised approaches, TCN and GRU, were experimented with using the datasets collected in the laboratory setting presented in Section 2. Thus, by varying the setup parameters (h, d) in {1.4, 1.6, 1.8, 2.0} × {N,NW,NE}, different accuracies were obtained, as reported in Figure 9 and Figure 10. As is visible from the two figures, in both cases, the best performance was achieved for h = 1.6 m and d = ‘N’.

**Figure 11:**
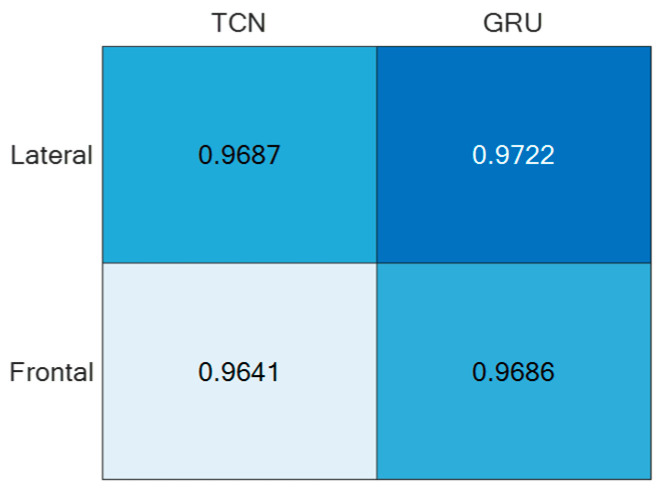
ADL recognition accuracies of the two supervised approaches under the direction d = ’N’ split into Frontal and Lateral.

**Figure 12:**
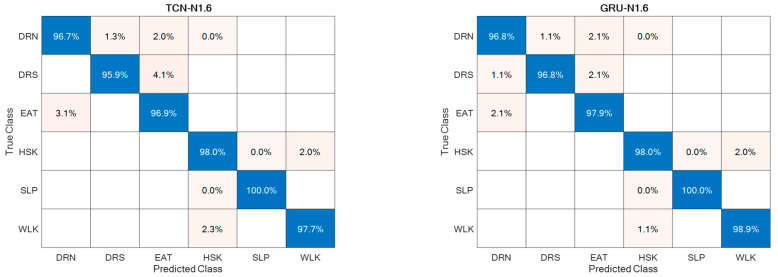
Confusion matrices for the best-performing setups.

Regarding the direction d = ‘N’, it was split into Frontal (captured from Cam 1) and Lateral (captured from Cam 2), thus estimating separately the corresponding accuracies, as reported in Figure 11. For the sake of completeness and due to space limitations, the confusion matrices are provided only for the best-performing setups in Figure 12.

**Figure 13:**
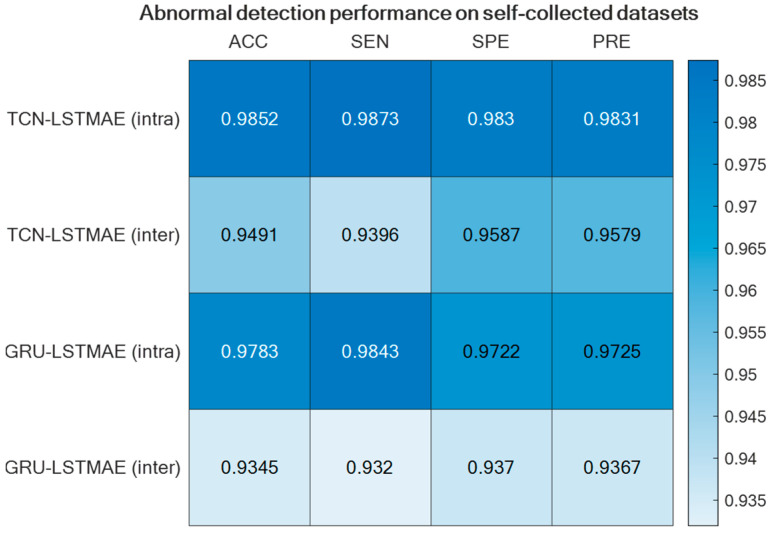
Abnormal gait detection performance for the two semi-supervised approaches, TCN–LSTMAE and GRU–LSTMAE, using either intra-class or inter-class training data.

**Figure 14:**
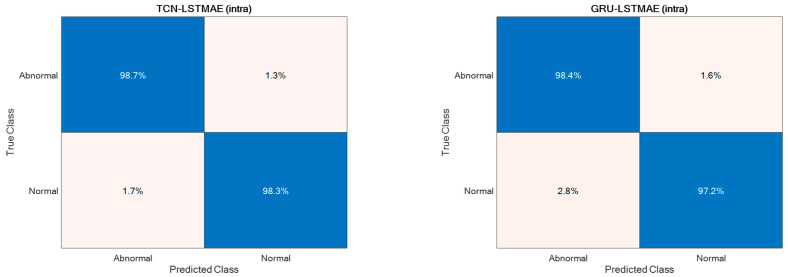
Confusion matrices of intra-class TCN–LSTMAE and GRU–LSTMAE for abnormal gait detection.

Regarding abnormal gait detection, the two semi-supervised approaches, TCN–LSTMAE and GRU–LSTMAE, were experimented with by using data collected in the laboratory setting and by using the best-performing setups previously defined for ADL recognition. In addition, each semi-supervised approach was trained using intra-class or inter-class data samples. The resulting performance in terms of ACC, SEN, SPE, and PRE are reported in Figure 13. In both semi-supervised cases, the best performance is obtained with intra-class training data, and the corresponding confusion matrices are reported in Figure 14.

**Figure 15:**
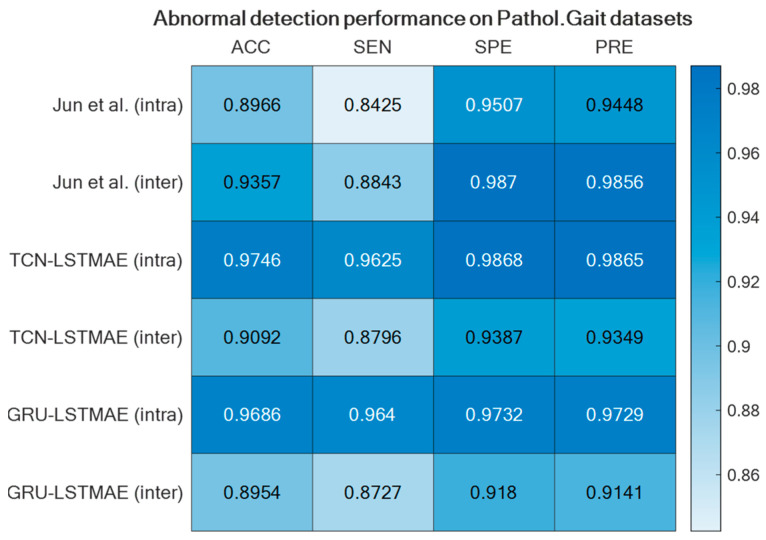
GRU-based approach by Jun et al. [11] compared with the two semi-supervised approaches using the Pathological Gait Datasets [11].

**Figure 16:**
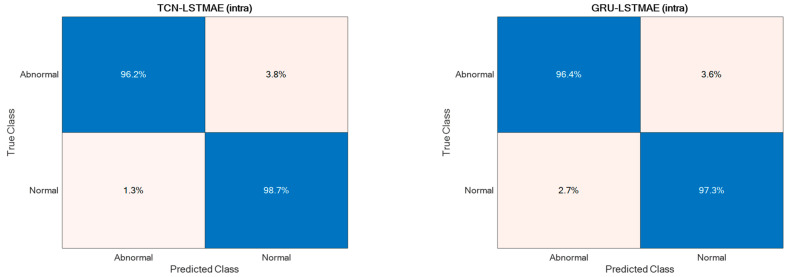
Confusion matrices of the two best-performing semi-supervised approaches.

For comparison reasons, the two semi-supervised approaches were also experimented with by using the Pathological Gait Datasets by Jun et al. [11]. Also, in this case, the best performance was achieved using intra-class training data, thus outperforming the original GRU-based approach presented by Jun et al. [11], as shown in Figure 15. The confusion matrices of the best-performing approaches, TCN–LSTMAE (intra) and GRU–LSTMAE (intra), are reported in Figure 16.

## 4. Discussion

The study presented in this paper focused on indoor monitoring for both ADL recognition and abnormal gait detection. In particular, the aim was to explore various camera setups and DL methodologies in order to find the most suitable setup for pursuing abnormal gait recognition during the monitoring of ADLs. 

Because the ADL monitoring was the entry-level functionality that the system should be able to provide, the two state-of-the-art supervised learning methodologies, TCN and GRU, and the camera setups were investigated for ADL recognition. 

For both of the supervised learning methodologies, the best-performing camera setup was achieved for height h = 1.6 m and direction d = ‘N’, i.e., when actions were performed frontally and laterally to the camera. However, the use of two (or more) cameras to capture the same scene may be cumbersome; thus, it is relevant to consider the employment of only one camera. For that reason, the best-performing direction d = ’N’ was separated into the contributions provided by the two cameras, which, due to the geometry of the experimental setup, viewed the scene frontally and laterally. And, as reported in Figure 11, the lateral view was that which provided the best accuracy with both unsupervised approaches. This aspect is of paramount importance in an indoor environment where no more than one camera can be deployed in each room. By appropriately positioning the camera, various ADL executions under different points of view can be captured over medium to long time intervals, among which the chances to include frontal or lateral views increase with the time.

The best-performing camera height of h = 1.6 m provided the smallest amount of noise in skeleton capture and tracking, even if that height may be uncomfortable to be maintained in indoor, home-like environments due to the susceptibility to occlusions. On the other hand, it should also be considered that the monitored subject is rarely left totally alone without any kind of supervision; thus, the caregiver can periodically check the correct camera operation. In addition, it is important to note that the aim of this study is not to detect dangerous situations, but to monitor for possibly medium–long time periods the execution of ADLs in order to detect abnormalities that may help with early diagnosis of neurodegenerative movement-correlated diseases and to monitor the evolution of such diseases.

The ADL recognition rates were quite high for both supervised approaches, with slightly better performance for the GRU-based one (Figure 12). This could be due to the superior ability of the GRU model to deal with long and articulated sequences, an especially helpful characteristic when actions are hierarchically structured, such as HSK, which is built on top of WLK with the addition of upper limb movements and torso rotations. In addition, from the point of view of abnormal gait detection, the ADL recognition performance was highly favorable, because actions most related to gait, i.e., WLK and HSK, were among those with higher recognition rates.

In this study, abnormal gait detection was investigated in close relationship with ADL recognition. The inclusion of various ADLs in the training dataset, instead of solely focusing on walking, facilitates the development of a model more reflective of real-life scenarios. In practical situations, individuals participate in many activities beyond mere walking. Enriching our training data with various ADLs is a deliberate choice to foster a model that demonstrates robust generalization to the intricate and diverse movements encountered in everyday life.

The rationale behind incorporating a range of ADLs is rooted in the recognition that restricting the training dataset to walking alone might lead to an overly specialized model that is less adaptable to the broader spectrum of everyday activities. By exposing the model to diverse ADLs, we seek to enhance its ability to discern abnormal gait patterns across different activities, thus ensuring its versatility and effectiveness in real-world applications.

The underlying assumption was that gait can be assessed during the execution of ADLs. For that reason, the state-of-the-art learning models for ADL recognition, TCN and GRU, were stacked with LSTMAE for abnormal detection based on the reconstruction error of learned features provided as inputs. However, while LSTMAE did not require labelled data, as it learned through an unsupervised modality, the features were supervised-learned by using TCN and GRU, which required labelled samples for training.

When addressing the challenge of abnormal gait detection, or abnormal event detection in a broader sense, obtaining labeled data for training supervised models proves to be highly difficult. Questions arise about the feasibility of observing abnormal gait—can it be realistically simulated in a laboratory setting, or must we await its manifestation in the monitored subject? Relying solely on observing the occurrence of enough anomalies to gather the necessary data (i.e., positive samples) for training a supervised model is evidently impractical.

Therefore, in this study, it was decided to investigate the approach based on semi-supervised training, i.e., based on the observation of normal gaits, to try to identify the abnormal gaits as deviations characterized by high reconstruction errors. However, normal gaits can be performed by the monitored subjects or simulated by other subjects (i.e., in a laboratory setting). In this study, both cases were investigated by training the semi-supervised model LSTMAE with inter-class and intra-class samples. Inter-class samples were TCN/GRU-learned features obtained from subjects different from the monitored ones, thus representing the simulation of normal gaits. On the other hand, intra-class samples were TCN/GRU-learned features obtained from the same monitored subject, representing a case in which normal gaits were observed directly from the monitored subject.

The abnormal gait detector, based on LSTMAE combined with TCN/GRU, provided better accuracies when trained with intra-class samples (Figure 13). This may be due to the fact that the normal gait of another subject differs substantially from that of the monitored subject, and this difference affects the reconstruction error, thus ultimately deteriorating the detection performance. Furthermore, unlike the case of ADL recognition, the performance of abnormal gait detection was slightly better when LSTMAE was combined with TCN rather than with GRU. 

At first glance, both TCN and GRU seem equally adept at processing time series data, with both architectures offering unique advantages. However, a closer analysis reveals nuances that may explain the performance gap observed. One primary aspect to consider is the memory utilization and sequence handling capabilities of both architectures. GRUs, despite their efficiency over traditional RNNs, do not possess extra memory units. They do operate similarly to LSTMs, and while they are designed to handle long-term dependencies, their intrinsic architecture might not be as efficient for capturing extensive historical sequences. On the contrary, TCNs are inherently designed to manage time series of any length by effectively mapping them to an output of the same length. This advantage becomes especially crucial when analyzing sequences where gait abnormalities may be subtly dispersed across a vast timeline.

Furthermore, when delving into the depths of these architectures—quite literally—the handling of deep structures offers more insights. Deep architectures can often run into the notorious problems of vanishing or exploding gradients. GRUs, though efficient compared to traditional RNNs, might still grapple with these challenges when dealing with long-term dependencies. TCN, with its incorporation of residual connections, provides a tangible solution to this problem. These connections ensure that even in the face of deep networks, the performance does not degrade, and gradient problems are kept at bay.

TCN’s flexibility further reinforces its advantage. Its combination of causal convolutions, dilated convolutions, and residual connections offers a malleable structure that can adapt to varied lengths and complexities of time series data. While GRU’s gating mechanism does introduce a degree of flexibility, its complexity might sometimes be an overkill for sequences that require simpler, more direct interpretations.

The performance superiority of the TCN-based abnormal gait detector was also confirmed by using the Pathological Gait Dataset [11]. In addition to comparing the approaches investigated in this study, the performance was also compared with the original GRU-based approach proposed by Jun et al. [11] on their datasets. Again, the performance of the combined TCN–LSTMAE approach was found to be superior to the GRU-based one (Figure 15 and Figure 16). It is also interesting to note that the GRU-based approach proposed by Jun et al. was trained using inter-class samples, i.e., obtained from observations of different subjects, but it was also trained using inter-class samples coming only from the same subject. However, in this case, the intra-class mode performed worse than the inter-class one, because the GRU-based approach proposed by Jun et al. [11] was a supervised one. The comparative results thus offer further confirmation of the superiority of semi-supervised approaches (i.e., TCN/GRU combined with LSTMAE) when trained in intra-class mode.

It is noteworthy that the supervised approach presented by Jun et al. [11] demonstrated superior performance in inter-class cases, as supervised models are specifically trained on abnormal samples. However, this approach poses practical challenges in real-life scenarios, as inducing or waiting for the occurrence of (rare) abnormal events for model training is impractical. In contrast, our semi-supervised model offers a more practical solution by being trained on normal events, which are abundantly observed in everyday activities. Abnormal events can then be identified by detecting deviations from normality.

Moreover, both supervised models, TCN and GRU, exhibited superior performance compared to state-of-the-art results, as reported by Climent-Perez et al. [15], in ADLs, such as drinking (87% accuracy in [15]), eating (87% accuracy in [15]), laying down/sleeping (86% accuracy in [15]), and walking (91% accuracy in [15]). This underscores the efficacy of our joint ADL–gait approach, thus showcasing improved gait abnormality detection without compromising the accuracy of ADL classification.

The dataset in our study involves data from three individuals, which prompts a closer examination of how this impacts the robustness of our model. Despite the limited number of participants, each individual performed every activity 12 times, resulting in a substantial dataset of 311,040 frames. This extensive repetition, combined with variations in three directions and four heights, contributes to a nuanced representation of the activities under analysis. For activity classification and abnormality detection, we utilized 30-frame sequences distinguishing between 6 classes and normal/abnormal activities, thus yielding a total of 10,368 instances. The dataset was meticulously divided into training, validation, and testing sets, which revealed consistent model performance across these partitions and indicated resilience to overfitting. While datasets with a larger number of individuals, such as the Pathological Gait Dataset [11], exist, the strength of our dataset lies in its careful curation and the richness derived from repeated activities under diverse conditions. The observed stability in model performance across varied scenarios reinforces its capability to generalize effectively.

In our pursuit of detecting abnormal gaits, particularly those associated with neurologically induced motor disorders, such as Parkinson’s and Huntington’s diseases, we aimed for a faithful simulation guided by the expertise of neurologists. The guidelines provided focused on replicating the nuanced characteristics of abnormal gaits by specifically addressing left–right asymmetry, tremor, rigidity, and postural instability.

It is crucial to highlight the distinctions of our approach compared to the study conducted by Jun et al. [11], as we performed a comparative analysis. In our simulation, participants were guided to emulate the characteristic features of neurologically induced motor disorders, resulting in gait abnormalities marked by left–right asymmetry, tremor, rigidity, and postural instability.

In contrast, the Pathological Gait Dataset curated by Jun et al. [11] featured five predefined pathological gaits—antalgic, stiff-legged, lurching, steppage, and Trendelenburg gaits. These predefined gaits provided a different perspective by focusing on specific pathological patterns rather than the broader spectrum of abnormalities encompassed in our neurologically guided simulation.

## 5. Limitations on the Use of Simulated Abnormal Gait

While our study employed simulated abnormal gaits guided by neurologists’ expertise, it is essential to acknowledge and discuss the inherent limitations associated with this approach. Simulating abnormal gaits, particularly those linked to neurologically induced motor disorders, such as Parkinson’s and Huntington’s diseases, introduces a set of challenges that warrant careful consideration.

Generalizability Concerns. Simulated abnormal gaits, by nature, may not capture the full spectrum of variability present in real-world pathological gaits. The controlled nature of simulations might limit the diversity of abnormalities observed in clinical settings. This raises concerns about the generalizability of our findings to the broader population affected by these disorders.

Complexity Replication. Accurately replicating the complexities of pathological gaits poses challenges. Neurologically induced motor disorders exhibit multifaceted symptoms, and our attempt to simulate left–right asymmetry, tremor, rigidity, and postural instability may not fully encapsulate the intricate nuances seen in clinical scenarios.

Variability Among Individuals. Individual variations in the manifestation of abnormal gaits further complicate the simulation process. While our study involved three individuals, the diversity in gait abnormalities observed across a larger and more diverse population may not be fully represented.

Ethical and Privacy Considerations. The simulation of abnormal gaits involves a balance between authenticity and ethical considerations. Real-world gaits may involve personal and sensitive aspects of individuals’ lives, and the ethical implications of simulating these abnormalities need careful attention.

In light of these limitations, it is imperative to interpret our results within the context of the simulated nature of the abnormal gaits. While our approach provides valuable insights and controlled conditions for analysis, the challenges outlined underscore the importance of further research involving clinical datasets and real-world gait abnormalities. Future studies should aim to validate findings in more diverse populations, considering the multifaceted nature of neurologically induced motor disorders. The use of simulated abnormal gaits serves as a pragmatic starting point for exploration, offering controlled conditions for analysis. However, researchers and practitioners must exercise caution when extending findings to real-world applications by recognizing the complexities and limitations inherent in the simulation process.

## 6. Conclusions

In this study, we have delved into gait analysis and its role in early detection and monitoring of neurological and musculoskeletal disorders. Our exploration centered on the automatic detection of abnormal gait utilizing 3D vision, with a specific emphasis on non-invasive data acquisition methods applicable in everyday environments. Our findings and the implications they hold are twofold, underlining their significance for both research and practical applications.

Firstly, we have examined various configurations for gait analysis, encompassing multi-camera setups deployed at different distances and angles, as well as performing daily activities in different orientations. Our investigation has further involved state-of-the-art DNN approaches, including TCN, GRU, and LSTMAE, thus revealing the superior performance of semi-supervised techniques when trained using the intra-class mode. We have also investigated diverse data representation formats, such as Euclidean-based representations, angular adjacency matrices, and rotation matrices. 

Secondly, our study underscores the relevance of integrating gait analysis with the monitoring of ADLs in the context of AAL applications. This holistic approach not only streamlines the monitoring process but also offers a comprehensive picture of an individual’s overall well-being. It presents a transformative shift from manual gait analysis, which is labor-intensive and expertise-dependent, to an automated, non-invasive, everyday monitoring system.

Looking ahead, we are actively pursuing ongoing work to initiate clinical trials involving hundreds of patients affected by movement-correlated neurological diseases, such as Parkinson’s disease and Huntington’s chorea. These trials will further validate the effectiveness and real-world applicability of our system by assessing its potential to enhance the lives of individuals suffering from such conditions.

## Figures and Tables

**Table 1:** Parameters of the experimental setup.

Parameter	Value/s
α	45°
b	5 m
h	{1.4, 1.6, 1.8, 2.0} m
Directions	{NE, N, NW}
ADLs	{EAT, DRN,DRS,HSK,SLP,WLK}
Volunteers	3

**Table 2:** Intel^®^ RealSense™ depth camera D435i technical specifications.

Parameter	Value/s
Non-ambiguity range	0.3–3 m
Depth technology	Stereoscopic
Minimum depth distance at maximum resolution	~28 cm
Depth sensor FOV	87° × 58°
Depth frame resolution	Up to 1280 × 720
Depth framerate	Up to 90 fps
RGB sensor FOV	69° × 42°
RGB sensor resolution	2 MP
Camera dimensions (Length × Depth × Height)	90 mm × 25 mm × 25 mm
Connector	USB-C 3.1 Gen 1

**Table 3:** Optimized parameters of the network architecture shown in Figure 5.

Network Parameters	Optimized Values
K	4
$N_{1},S_{1},D_{1}$	128, 8, 0.5475
$N_{2},S_{2},D_{2}$	128, 5, 0.1491
$N_{3},S_{3},D_{3}$	128, 13, 0.005
$N_{4},S_{4},D_{4}$	128, 8, 0.6214

**Table 4:** Optimized parameters of the LSTMAE using activations from TCN.

Network Parameters	Optimized Values
$B_{1},F_{1},D_{1}$	256, 200, 0.0083
$B_{2},F_{2},D_{2}$	128, 100, 0.2875
$B_{3},F_{3},D_{3}$	256, 200, 0.0095

**Table 5:** Optimized parameters of the LSTMAE using activations from GRU.

Network Parameters	Optimized Values
$B_{1},F_{1},D_{1}$	256, 200, 0.0106
$B_{2},F_{2},D_{2}$	128, 100, 0.3127
$B_{3},F_{3},D_{3}$	256, 200, 0.0112

## Data Availability

Data are available upon request due to privacy restrictions.
